# Platelets Extracellular Vesicles as Regulators of Cancer Progression—An Updated Perspective

**DOI:** 10.3390/ijms21155195

**Published:** 2020-07-22

**Authors:** Magdalena Żmigrodzka, Olga Witkowska-Piłaszewicz, Anna Winnicka

**Affiliations:** Department of Pathology and Veterinary Diagnostics, Institute of Veterinary Medicine, Warsaw University of Life Sciences (WULS-SGGW), Nowoursynowska 159c, 02-787 Warsaw, Poland; olga_witkowska_pilaszewicz@sggw.edu.pl (O.W.-P.); anna_winnicka@sggw.edu.pl (A.W.)

**Keywords:** extracellular vesicles, exosomes, ectosomes, neoplasia

## Abstract

Extracellular vesicles (EVs) are a diverse group of membrane-bound structures secreted in physiological and pathological conditions by prokaryotic and eukaryotic cells. Their role in cell-to-cell communications has been discussed for more than two decades. More attention is paid to assess the impact of EVs in cancer. Numerous papers showed EVs as tumorigenesis regulators, by transferring their cargo molecules (miRNA, DNA, protein, cytokines, receptors, etc.) among cancer cells and cells in the tumor microenvironment. During platelet activation or apoptosis, platelet extracellular vesicles (PEVs) are formed. PEVs present a highly heterogeneous EVs population and are the most abundant EVs group in the circulatory system. The reason for the PEVs heterogeneity are their maternal activators, which is reflected on PEVs size and cargo. As PLTs role in cancer development is well-known, and PEVs are the most numerous EVs in blood, their feasible impact on cancer growth is strongly discussed. PEVs crosstalk could promote proliferation, change tumor microenvironment, favor metastasis formation. In many cases these functions were linked to the transfer into recipient cells specific cargo molecules from PEVs. The article reviews the PEVs biogenesis, cargo molecules, and their impact on the cancer progression.

## 1. Introduction

The number of research work and scientific papers that discuss the involvement of cell-derived extracellular vesicles (EVs) in multiple physiological and pathological processes has increased rapidly during the last two decades. EVs might have an influence on target cells by delivering ligands and signaling complexes, and transferring mRNA and transcription factors that cause the epigenetic reprograming of recipient cells. EVs are submicron spherical membrane bound structures, that are generated by different prokaryotic (termed as membrane vesicles) and eukaryotic cells [1,2,3]. EVs nomenclature take into account their cellular origin and size. Their size ranges between 10 nm to 5 µm and comprises three heterogeneous populations of vesicles—exosomes (EXSMs), ectosomes (ECTSMs) also named microparticles (MPs), and apoptotic bodies (ABs) [4,5]. EVs actively secreted form parental cells with a diameter of 10 to 100 nm are named EXSMs, and those with a diameter ranging between 100 nm to 1 µm are ECTSMs. Lipid bilayer membrane protects their cargo from enzymes like proteases and ribonucleases [6]. The largest of EVs are ABs (with diameter 1–5 µm) represented by clumps of material generated during the late stage of cell apoptosis [5,6,7].

During activation, maturation, proliferation, stress, aging, or apoptosis, cells shed EVs into the extracellular space [8]. Their presence in a number of body fluids including—urine, synovial fluid, bronchoalveolar lavage fluid, saliva, and bile was confirmed [7,9,10,11]. In the bloodstream, EVs are released by—erythrocytes, leukocytes, platelets (PEVs), megakaryocytes, and endothelial cells [10,12]. In addition, EVs are also secreted by cancer cells known as tumor-derived extracellular vesicles (TEVs) [4,12]. In both healthy subjects and those with a variety of pathologies, peripheral blood is a rich source of EVs, where the most abundant population are PEVs. Their percentage ranges between 70 to 90% of all EVs in the plasma of healthy individuals [13,14,15].

In 1967, Peter Wolf described “platelet dust”—a subcellular material derived from thrombocytes in the plasma and serum of healthy individuals [16,17]. This was a milestone in medicine research, allowing further examinations evaluating PEVs involvement in physiological and pathological processes. PEVs share many functional features with PLTs. These tiny fragments smaller than platelets (PLTs) were secreted during PLT activation and were known to be crucial in coagulation and clot formation [16,18]. Despite the fact that PLTs play a crucial role in hemostasis, PEVs coagulation capacity is several dozen higher than PLTs [19]. Platelets microparticles (PMPs) are enriched in tissue factor (TF), coagulation factors, and dozens of them expose about 3-fold higher phosphatidylserine (PS) concentration on the outer membrane than PLTs [20]. The coagulation process initiated by TF connection with coagulation factor VII, activates coagulation cascade. Activated PLTs, PMPs PS + offer a catalytic surface for the coagulation and binding of consecutive clotting factors. Moreover, in healthy individuals, the presence of integrin αIIbβ3 (CD41/CD61) on PMPs supports fibrin clot formation [21]. In various bleeding disorders, abnormalities in PMPs functions and their reduced number in blood were reported [22]. On the other hand, their increased amount was presented in thrombotic state and other pathologies [23]. PLTs of patients described by Castaman are unable to shed PMPs, conversely to patients with Scott syndrome in which the PMPs number is adequate, but the incorrect translocation of PS impairs prothrombinase activity, and causes hemorrhagic diathesis [22]. Patients with immune thrombocytopenia have higher PEVs level than healthy individuals, which might be an evolutionary way to prevent blood loss and maintain tissue integrity [24]. Additionally, contemporary papers showed that PEVs might be a potential biomarker or prognostic factor in other pathologies—inflammatory, cardiovascular, and autoimmune diseases, solid tumors and hematological malignancies [14,25].

In this review, the role of PEVs in the cancerogenesis, tumor growth, and metastasis formation in distant organs is reported. Furthermore, the possible evaluation of PEVs as markers for cancer detection, and effectiveness of anticancer treatment is discussed.

## 2. EVs Biogenesis and Elimination

Based on the current knowledge, the mechanism of EVs formation and secretion to the extracellular space vary, depending on the EXSMs or ECTSMs descent. The EXSM definition was originally used for microparticles secreted from variety of cultured cells, thereafter, Johnstone and colleagues in 1987 explained the mechanism of transferrin receptor loss during reticulocytes maturation via secretion of nanosize vesicles; for this term EXSMs is used [26]. The latest research confirmed that the pathways of EVs biogenesis might differ between the parental cells types and EVs secretion, which does not seem to be accidental [1,27].

### 2.1. ECTSMs Formation

**Figure 1:**
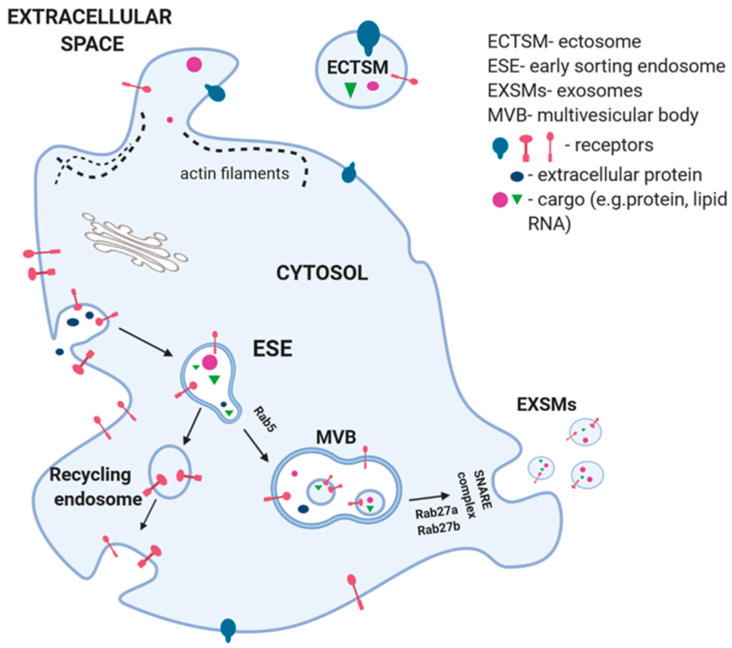
Extracellular vesicle biogenesis and secretion. The exosomes (EXSMs) generation begins with the membrane bulging into the lumen of the ESE. Part of them form a part of the plasma membrane (recycling endosome), others are converted into multi vesicular body (MVB). Members of the Rab family, Rab27a and Rab27b, are involved in MVB transport and fusion with cell membrane. Transmembrane protein complex SNARE enables the MVB to dock with the cell membrane that leads to release of EXSMs to extracellular space. Ectosome (ECTSM) are formed directly by cell membrane blebbing. This process is initiated with an increase in intracellular calcium that causes the activation of enzymes—floppase and scramblase and the inhibition of flippase. This causes the rearrangement of phospholipids in the cell membrane, as well as results in breaking bonds between cytoskeleton and partial degradation of actin filaments. During formation of EXSMs and ECTSMs, mRNA and miRNA that are located in cytoplasm are randomly entered.

The blebbing of the plasma was documented in apoptosis during ABs formation, but it was confirmed as well in ECTSMs biogenesis. Changes in lipid components affect the rearrangement within plasma membrane. This process is initiated by an increased level of intracellular calcium ions. It causes activation of floppase and scramblase enzymes and inhibition of flippase (Figure 1) [1,8]. The membrane phospholipids—PS and phosphatidyl-ethanolamine, are vertically translocated from the inner leaflet to the outer cell membrane surface. The rearrangement breaks the bonds between cytoskeleton and cell membrane phospholipids. Partial degradation of actin filaments leads to restructuring of the cytoskeleton filaments, which favor formation of ECTSMs [1,8,10].

The fast phospholipid membrane remodeling and PS exposure are relevant for PLTs physiological procoagulant response in hemostasis. PMPs formation in the circulation could result from PLTs activation via multiple agonists, high shear stress or apoptosis [20,28]. In the high shear rate, the loss of membrane integrity is initiated through the dislocated connection between the membrane glycoprotein Ib receptor (CD42b) and PLTs cytoskeleton, which began PMPs formation [20]. Natural PLTs activators, such as thrombin or collagen, induce PMPs formation via transmembrane integrin receptor gpIIb/IIIa (CD41/CD61) or tetraspanin 29 [29]. Altogether, these observations become the starting point for subsequent works assessing, how different types of PLTs activators induce PMPs formation, and how they affect the heterogeneity of PEVs population. Noticeably, a research conducted in 2017 confirmed that PS negative tubular PMPs population with structural similarities to filopodia could be formed during PLTs activation. Lack of PS expression on their surface implied that during their formation, there is no PS translocation [30].

### 2.2. EXSMs Formation

EXSMs generation begins with the inward bulging of the plasma membrane by endocytosis into the cytoplasm lumen. It leads to forming early sorting endosomes (ESEs) (Figure 1) [1]. Part of ESEs is returned into plasma membrane, other under the Rab5 control are changed into late endosomes or multivesicular bodies (MVBs) [1,10]. During this process, proteins and antigens are packaged into intraluminal vesicles (ILVs) and the budding of the ESEs membrane transform into MVBs [31]. Four protein subunits of the endosomal sorting complex required for transport (ESCRT) machinery are involved in this process. ESCTR-III is essential for the scission of ILVs into MVBs lumen. Cargo clustering and membrane budding can occur by ESCRT-dependent or -independent machinery [1]. ESCRT-0 recognizes ubiquitinated proteins (cargo) by the hepatocyte growth factor-regulated tyrosine kinase substrate (Hrs), in association with clathrin. This complex helps ESCRT I and II to connect with ESCRT 0 and ubiquitinated cargo, on the part of the endosomal membrane, where it will finally pullulate. ESCRT III connects with the complex and ultimately bud ILVs into the endosome [32]. The MVBs fuse with the plasma membrane to secrete the ILVs as exosomes or absorb with lysosomes for their degradation [1]. Members of Rab family, Rab27a and Rab27b, are essential mediators in transport of MVBs and its fusion with cell membrane. Transmembrane protein complex SANRE enables dock EXSMs with cell membrane that leads to the release of EXSMs to extracellular space (exocytosis). Increased concentration of calcium ions is one of the EXSMs secretion regulators [33]. Targeting selected Rabs via specific inhibitors modulates their structure or secretory function and becomes a new promising strategy of limiting EXSMs formation, both by PLTs and cancer cells. Wang et al. showed in a pre-clinical study, that elevated number of PMPs in patients with sepsis after intravenous administration of small GTPase inhibitor NSC23766, reduced PMPs secretion for about 87% [34,35].

Aatonen et al. showed that PMPs and platelet derived EXSMs (PdEXSMs) biogenesis is also observed by non-activated PLTs [36]. Examination potency of various agonists on EVs formation confirmed that Ca^2+^ ionophore is the strongest agonist, these include—thrombin, collagen, LPS, TRAP-6, and the weakest one is ADP [36]. Moreover, authors considered that Ca^2+^ ionophore causes vesiculation in unselective way or fragmentation and ABs formation, and should be advisedly used as agonist. The strongest PdEXSMs activators are thrombin and collagen or collagen-related peptide XL. Interestingly, the proteins cargo in PdEXSMs derived from stimulated PLTs was richer than from resting PLTs [36]. Nowadays, the utility of EXSMs as a new diagnostic cancer marker is extensively studied. Recent work performed by Lea et al. showed an increased number of EXSMs with PS expression in peritoneal fluid and plasma of patients with ovarian carcinoma [37]. It confirmed that, when PS is routinely used as a PMPs marker, it is also present on cancer derived EXSMs and causes a possibility to exploit these results in early diagnostic tests of women with ovarian malignancies [37].

### 2.3. EVs Elimination and Impact of Storage Conditions on PEVs Number

The PEVs rapid clearance from circulation varied depending on their molecular content, and the induction signal in different species [38,39]. As they have pro-coagulant and pro-inflammatory nanosize structures, their rapid turnover is essential for prevention of thrombotic diseases. PMPs turnover in rabbits is less than ten minutes, compared to people where PMPs were shown in circulation for more than 3 h [20,38,39]. Flumenhaft found that mice PMPs are eliminated from bloodstream within 30 min [40]. PEVs could be phagocytized after their opsonization with thrombospondin or complement components C3b [40]. The PS on the PMPs outer leaflet of the plasma membrane is recognized by macrophages and it originates a signal to remove them. Moreover, the role of lactadherin (LA) in clearance of EVs from circulation is discussed [41]. LA secreted by macrophages and adipocytes is also detected on the circulating PMPs. An increased PEVs level was observed in lactadherin–deficient mice, which could suggest the role of LA as a one of “eat-me” signals for phagocytosis [41]. Dasgupta et al. showed that developmental endothelial locus–1 in endothelial cells mediates PS-positive PMPs elimination via endocytosis [17,42]. Shorter half-life of ECTSMs, compared to EXSMs in blood, might arise from the higher concentration of membrane lipids in ECTSMs and activity of phospholipase A2 in serum [43]. Furthermore, EXSMs elimination via IgM immunoglobulins binding to lipid lysophosphatidylcholine was reported and liver macrophages were shown to be crucial elements of EXSMs clearance [43,44].

In EVs analysis, preanalytical steps standardization is crucial for the minimization of false results of PEVs number and their quality tests. Different anticoagulants could activate PLTs during blood collection and storage. Wisgrill et al. confirmed that the EVs number and their functionality is stable in sodium citrate for 8 h in room temperature (RT), after blood samples collection [45]. In EDTA, routinely used in clinical practice, PMPs and erythrocytes′ derived EVs count is stable for 48 h in RT [45]. Thus, it could be an alternative when the collected samples are stored before analysis [45].

## 3. Content of Platelet Extracellular Vesicles

Physiological or pathological processes in parental cells define their EVs cargo and biological properties. As described above, the PEVs formation, membrane composition and specific markers expression on the outer membrane leaflet depends on the PLTs activators (Ca^2+^ ionophore, adenosine diphosphate, thrombin, collagen, epinephrine) [20]. Most of the EVs circulating in plasma are classified as PEVs based on their surface receptors. Nevertheless, heterogeneity of PEVs surface receptors starts discussion about EVs derived from megakaryocytes (Mk-EVs), as a part of PEVs subpopulation [46]. The EVs phenotyping conception to distinguish PEVs from Mk-EVs involves the usefulness of cluster of differentiation (CD) CD41/CD61 as a constitutive marker for both PLTs and Mks, while CD62P and CD107a act as a PLTs activation markers [47]. Flaumenhaft et al. showed that mouse and human Mk-EVs are PS/CD41/CD61 positive and CD62/CD107a negative [46]. In support of this finding, after irradiation of bone marrow, the CD61 positive EVs population largely disappears from mice circulation, whereas CD62P remains unchanged [48]. A study by Brisson et al. showed that small PMPs population—PS negative and CD41 positive, is a result of cell membrane shedding without PS redistribution. Moreover, PMPs could contain organelles like mitochondria and dense granules [30]. EVs are identified based on their size and expression of characteristic surface markers. PS expression is an a ECTSMs marker, when the presence of tetraspanin CD63 is used for EXSMs identification. During PLTs activation, both ECTSMs and EXSMs are CD63 positive but the CD63 expression is higher on EXSMs. It could be useful for determining the purity of the EXSMs population [30,49]. A characteristic of PEVs is the diversity of their surface markers and cargo. PEVs display a wide array of bioactive molecules like adhesion molecules, chemo- and cytokines, apoptosis regulators, miRNAs. They also harbor a broad spectrum of coagulation factors, enzymes, complement proteins, and bioactive lipids (Table 1). PEVs express glycoprotein (gp) IIb/IIIa, Ib, IIa, as well as P-selectin and a lysosome-associated glycoprotein-1 (LAMP-1). C-type lectin domain family 1-member B (CLEC-2) and gp VI expression was documented on Mk-EVs [17]. PdEXSMs are substantial with proteins from α granules, whereas ECTSMs are substantial with lipid mediators and mitochondrial proteins [17,50].

Molecules presented on PEVs were involved in triggering receptors on the target cells or regulating them via bioactive molecules, signaling molecules or a plethora of genetic material including miRNAs [51]. PEVs can interact with donor cells in multiple ways—(i) stimulation via signaling complex, using specific PEVs surface receptors and lipids; (ii) transfer membrane receptors and adhesion molecules; (iii) horizontal transfer of heterogeneous proteins, miRNAs, bioactive lipids, and other factors including infectious particles (prions) or even organelles (mitochondria) [3].

PEVs are able to transfer receptors expressed on their surface (i.e., CD41, CD61, CD184, CD62P, PAR-1) to recipient cells (monocytes, myeloblasts, hematopoietic stem cells) and induce their adhesion or proliferation [3,27]. PEVs functional gpIIb/IIIa (CD41/CD61) transferred to neutrophils, activated NF-κB, in response to GM-CSF and enhanced inflammation [77]. Tang and colleagues showed that PEVs transfer arachidonate 12-lipoxygenase to mast cells, which increased synthesis of one of the negative regulator of inflammation lipoxin A4 (LXA4) [27,55]. Thus, PEVs play both positive and negative role in inflammation response, depending on the target cell.

PEVs are rich in sphingosine 1-phosphate, metalloproteinases, heparyanase, PDI, and arachidonic acid (AA) [3]. Transfer of AA by PMPs to monocytes and endothelial cells induced by prostanoids and cyclooxygenase 2 synthesis enhances these cells interactions [3]. Treatment of human umbilical vein endothelial cells (HUVECs) with PEVs showed intensification of angiogenesis and cell proliferation versus activated charcoal treated PEVs (removed nonpolar lipids), where a reduction of these effects was observed. This experiment showed that PEVs lipid components were involved in HUVECs stimulation [27,78]. The horizontal transfer of non-coding RNAs via EVs regulates gene expression by post-transcriptional repression. miRNA from parental cells encapsulated in EVs was protected from ribonuclease activity in circulation [51]. In human’s, several PMPs miRNAs were detected, e.g., miR-19, miR-21, miR-22, miR-126, miR-133, miR-146, miR-185, miR-223, and miR-320b [3]. Moreover, it was confirmed that PEVs miRNA was transferred to macrophages, endothelial, and cancer cells. In macrophages, MiR-126–3p transferred from PEVs led to decreased ATF3 and ATP1B1 expression and protein synthesis [27]. Recently presented data support the notion of PEVs tumor microenvironment infiltration and interaction with cancer cells via the mechanisms described above.

## 4. PEVs in Cancer Progression

PEVs are highly interesting group of EVs because of their percentage participation in bloodstream, as well as their increased number in patients with cancer, such as glioblastoma, gastric, lung and skin cancer, and other diseases. This makes them potentially useful as a diagnostic marker [79]. It is known that PLTs facilitate cancer metastasis. Moreover, the number of papers that discuss PEVs contribution in cancerogenesis increased recently [51,80]. EVs as cell-to-cell messenger molecules can start phenotypic and functional changes in donor cells, by reaching the recipient cells and delivering EVs content. PEVs are also discussed as potentially early markers of disease progression.

### 4.1. PEVs in Tumor Angiogenesis

The cancer cells without blood circulation can grow up to 2 mm^3^ in diameter, forming a tumor and then stop and undergo apoptosis or necrosis [81]. Growth of the vascular network is pivotal for the cancer cells survival, proliferation, as well as metastatic spread of cancer [81]. Angiogenesis is essential for formation of a new vascular network that supplies nutrients, oxygen, and immune cells, and also removes waste products of cellular metabolism. Therefore, angiogenesis is a critical factor in the progression of cancer. The tumor microenvironment (TME) consists of diverse cellular populations, including tumor cells, endothelial cells, fibroblasts, infiltrating immune cells (monocytes, macrophages, neutrophils, mast cells, T cells), extracellular matrix, and newly formed blood vessels [79]. The PEVs interaction with TME components could reveal their functions in cancer progression. Newly stirring blood vessels permit tumor growth, which is critical in cancer progression. Interestingly, Happonen et al. demonstrated a mechanism of PEVs transfer to human aortic endothelial cells (HAECs) and HUVECs [82]. PS-positive PEVs are taken up by phagocytosis via tyrosine kinase receptor Axl, and its ligand protein Gas6 on endothelial cells [82]. Janowska-Wieczorek et al. used lung cancer cell lines to elucidate PEVs importance in cancer angiogenesis [83]. After PEVs stimulation of IL-8 (about 35-fold), vascular endothelial growth factor (VEGF) (3-fold) and scatter factor (4-fold) mRNA expression increased in the A549 cell line [83].

PEVs delivery of bioactive molecules like cytokines or microRNA to recipient cells could regulate tumor growth [84]. miRNAs are small non-coding RNAs that regulate gene expression post-transcriptionally. Anene et al. demonstrated regulatory angiogenesis miRNAs transfer from PEVs to HUVECs cells during co-culturing on extracellular matrix gel [85]. A robust capillary-like structure formation and simultaneously decreased synthesis of anti-angiogenic thrombospondin-1 (THBS-1) was observed. miRNA Let-7 a from PEVs was delivered to HUVECs and targeted THBS-1 mRNA to induce angiogenic responses of HUVECs [85]. Blood vessel formation is controlled by a balance between localized production of pro- and anti-angiogenic molecules and changes in THBS-1 concentration is the key determinant of this “angiogenic switch” [85]. PEVs ability to bind TF and the platelet-activating factor potentiates their pro- angiogenic competence even more [86].

Pan et al. demonstrated that after incubation, PEVs with HUVECs cells miR-223 level in endothelial cells increased, which promoted glycation end-product-induced vascular endothelial cell apoptosis via targeting insulin-like growth factor 1 receptor [87]. Another work showed that HUVECs cells preferentially uptake miR-223 from PEVs generated by thrombin-activated PLTs [4,88]. This leads to the formation of functional Argonaute 2 (Ago2) miR-223 complexes. These complexes are able to regulate gene expression and protein level for ephrin A1 and F-box/WD repeat-containing protein 7 in HUVECs cells and conduces apoptosis [4].

Increased angiogenesis in TME could be a result of metalloproteinase-1 (MMP-1) transfer, as well as increased MMP-9, VEGF, and IL-8 mRNA expression in lung cancer cells lines, after co-incubation with PEVs [83]. Moreover, PEVs molecules from α granules like VEGF, platelet-derived growth factor and fibroblast growth factor are a component of their cargo with pro-angiogenic properties.

### 4.2. PEVs in Migration, Invasion, and Metastasis

A key for distant metastases formation is cancer cells passage through the newly formed vascular walls in primary tumor, surviving in the circulation, and finally proliferation at the distant tissue. In solid tumors, vasculature is highly permeable, allowing the possibility to PEVs infiltration to TEM and contact with cells. A great number of studies indicate the PEVs involvement in cancer progression and some discuss their anti-cancer properties. Michael et al. showed that PEVs have the ability to infiltrate murine and human tumors [84]. This ability creates conditions for the horizontal transfer of miRNA-24, which targets mitochondrial mt-Nd2, and Snora75. This entails mitochondrial dysfunction and results in an increased cancer cell apoptosis [84].

Bakewell and colleagues showed that platelets gpIIb/IIIa antagonists minimize formation of distant metastasis from B16 melanoma cells in bones, due to the inhibition of the interaction between cancer cells and PLTs [89]. Lung cancer cell line A549 increases adhesiveness to the fibrinogen and HUVECs, after receiving CD41 from PEVs. PEVs chemoattract lung cell lines from 2.5 to 7-fold more than the control [83]. Moreover, evaluation of PEVs interaction with lung cancer cell lines confirmed the activation of mitogen-activated protein kinase (MAPK) MAPK p42/44 and AKT, signaling pathways participating in proliferative responses [83]. Murine lung cells covered by PEVs injected intravenously into mince resulted in significant increase metastasis formation in lungs and bone marrow [83]. Transfer onto the surface of donor cells CD184-, a chemokine receptor type 4 from PEVs and respond to stromal cell-derived factor 1, which is rich niche in bone marrow in the murine model, confirmed their high metastatic potential [83]. Moreover, activation of cyclin D2 by PEVs in lung cancer cell lines could change the phenotype of cancer cells into a more invasive phenotype. Similar observations were made in human squamous carcinoma or breast cancer cell lines in murine in vivo model [90].

Interestingly, Gasperi and colleagues confirmed the modulatory influence of polyunsaturated fatty acids (PUFAs) diet, especially the ω3 and ω6 on cellular processes in carcinogenesis [62]. The PUFAs ω3 cancer preventive activity is well known, in contrast to high concentration of ω6 in diet, which correlates with higher risk of breast and prostate cancer [62,91]. Their role in cancerogenesis is related to changes in fatty acids compositions of membrane rafts in cells membranes. PEVs contains miR-126 and miRNA-223, which are important players in tumorigenesis. VEGF-dependent proliferation of endothelial cells is stimulated by miRNA-126, while miRNA-223 inhibit formation of new blood vessels by targeting endothelial β1 integrin [92]. Gasperi et al. examined the influence of increased level of PUFAs ω6 on both PEVs formation and their cargo [62]. The newly formed PEVs had an increased amounts of miRNA-123 and miRNA233. Breast cell line BT549 blocked its cell cycle and decreased cell migration after internalizing PEVs [62].

A Tang et al. study revealed an important PEVs role in the epithelial-to-mesenchymal transition of ovarian epithelial cancer cell line (SKOV3). miR-939 transfer leads to enhanced invasion and cancer progression [93,94]. Tropomyosin 3 (TPM3) contributes cancerogenesis in thyroid papillary carcinoma and esophageal squamous cell carcinoma by fusing neurotrophic receptor tyrosine kinase 1 and PDGF receptors [95]. Yao et al. demonstrated increased TPM3 mRNA in PLTs and revealed their transfer by PEVs into breast cancer cells and promotion of an invasion [94]. Moreover, in patients with distant metastases, compared to subjects without metastases TPM3 mRNA in PLTs was significantly increased [94].

Another interesting issue is the ability of cancer cells to educate PLTs. Zarà et al. demonstrated that breast cancer cell lines—highly aggressive MDM-MB-231 and MCF7 could educate PLTs to produce PEVs in an amount similar to that after thrombin activation [96]. Next, those PEVs were co-cultured with cancer cells to investigate if the newly formed PEVs impact cells. Only in the MDM-MB-231 cell line, authors observed cells activation and phosphorylation of p38MAPK and myosin light chain. Moreover, increased migration and invasion was noted. This experiment showed that PEVs can novel paracrine-positive feedback mechanism initiated by MDA-MB-231 to escalate their invasive phenotype [96].

PEVs formed by PLTs during apoptosis-like process show surface gpIIb/IIIa, and PS and stimulate their own phagocytic removal by monocytes, moreover, they are able to change macrophages into M2 macrophages [97,98]. In contrast to effect on endothelial cells, after PEVs miR-223 transfer into gastric cell line SGC7901, increased proliferation and invasion in vitro, as well as decreased apoptosis, was observed. This showed that horizontal miRNA transfer via PEVs could have diverse effect contingent on donor cells [4,99]. Another noteworthy experimental work showed that peripheral blood mononuclear cells (PBMCs) isolated from patients with B-precursor acute lymphoblastic leukemia had increased apoptotic markers CD95, active caspse-3, and an increased number of apoptotic cells, after two days of co-culturing with PEVs [100].

Cancer cells transmigration from circulation into the tissues is mediated likewise by tissue-specific enzymes, the majority of which belongs to the MMP family. Dashevsky et al. confirmed transfer of MMP-2 and its′ increased secretion from Cl-1 cells after co-culturing with PEVs. Interesting observation was made when Cl-1 cells were incubated with PEVs lysate. Values of MMP-2 concentration and secretion were similar to that after cells co-culturing with PEVs. It suggests that the transfer of MMP-2 is not dependent only on PEVs internalization. The other possible candidates for increased MMP-2 value might be free miRNA from PEVs lysate or lysophosphatidic acid (LPA) as an MMP-2 activator presented on PLTs and in prostate cancer cells [101].

Natural killer (NK) cells efficiently recognize and kill circulating tumor cells of almost any origin, but their effectiveness in TME is discussed. PEVs miR-183 transfer into NK cells suppressed activator adapter DAP12 and suppressed their cytolytic functions in tumor-associated NK cells [102]. PEVs could also horizontally transfer functional miR-126–3p into primary human macrophages. The PEVs dose-dependent down regulation of miR-126–3p targets CCL4, CSF1, and TNF was observed. Decreased secretion of cytokines/chemokines was correlated with reprogramming into phagocytic macrophages [88,103]. The role of TF in angiogenesis and metastasis formation is well documented, therefore, the role of TF-positive PEVs in tumor growth seems clear. Another interesting aspect of PEVs as a potentially important immune checkpoint in cancer biology is a presence of PS on PEVs surface. PEVs as an abundant source of PS might be a possible ligand for PS receptor (PSR) on the immune cells. Activation of PS–PSR pathway leads to the inhibition of innate and adaptive immune response in TME, as well as in circulation [104]. The new oncotherapy strategies examined the PSR inhibitors as a new anticancer target, but only a highly selective inhibition strategy could be applied in the cancer treatment. Table 2 summarize PEVs pivotal role in crosstalk between PLTs and other cells, particularly with cancer cells (Table 2).

## 5. The Potential of PEVs as Diagnostics Cancer Biomarkers

PEVs number in blood was raised about twice in myeloproliferative neoplasms, compared to healthy controls, up to four times in oral cancer and colorectal subjects and more than ten times in breast cancer patients [86,106]. The highest concentration of PEVs, more than 30-fold, was noticed in patients with IV stage of gastric cancer. In each group, the highest PEVs concertation were demonstrated in advanced cancer stages and in patients with distal metastases [86,106,107,108].

Investigation in patients with non-small cell lung cancer (NSCLC) categorized based on disease progression, showed the significantly higher number of circulating EVs from activated or apoptotic PLTs and from endothelial apoptotic cells, compared to healthy subjects. Changes in EVs levels in different stages of NSCLC showed that serial measurements of circulating PEVs are valuable prognostic biomarkers, mainly in the advanced stages of NSCLC [109].

PEVs as source of anionic phospholipids and TF on their surface are one of the important factors of procoagulant activity. Data demonstrated by Ren et al. showed the significantly increased number of EVs and PEVs in patients with oral squamous cell carcinoma (OSCC) in peripheral blood. PEVs level was also positively correlated with clinical stage and with fibrinogen concentration and patients hypercoagulable state [107]. Mege and colleagues showed correlations between increased PEVs number and the stage of the disease in patients with pancreatic cancer and colorectal cancer. They suggested that PEVs concentration in blood could be a useful marker for evaluation of the disease progression in these types of neoplasia [110].

Yenigürbüz et al. described another aspect of increased PEVs number in patients with neoplasia. Thromboembolism is one of the complications during induction of therapy in pediatric acute lymphoblastic leukemia (ALL) patients [111]. Children with ALL have increased levels of ABs, PEVs, endothelial-derived, and tissue factor-positive microvesicles during induction therapy. Further studies are needed to confirm the PEVs contribution in thromboembolism during the induction therapy period in children with ALL [111]. Similar observations were made in adult patients with myeloproliferative neoplasia, where the number of TF positive PMPs and endothelial derived EVs was significantly increased, which might also play a role in thrombotic complications in that group of subjects [112]. Tjon-Kon-Fat et al. demonstrated that tumor educated PLTs are a source of prostate cancer biomarkers [113,114]. In this context it seems to be interesting to evaluate the presence and role of EVs generated from tumor-educated PLTs.

## 6. The Potential of PEVs in Cancer Therapy

The paradigm of using nanoparticulate pharmaceutics as delivery vectors was established over the past decade [56]. To use EVs as drug transporters, their pharmacokinetics should be analyzed. Mice models of EXSMs distribution showed that the route of administration, EXSMs origin, and concentration critically influenced their biodistribution [115]. In the mice model, after intraperitoneal and subcutaneous administration of EXSMs, they preferentially localized in the pancreas and gastrointestinal tract. Whereas, intravenous administrated EXSMs were detected in the spleen and the liver [116]. In addition, EXSMs loaded with therapeutic anti-miRNA could be transferred locally into tumor or systemically. Other therapeutic strategies in cancer therapy were elimination of EXSMs from blood or prevention of EXSMs fusion with target cell [117,118]. Various strategies of using EXSMs in anticancer therapy are characterized in the literature, but more research is still needed.

In an elegant study, Kailashiya et al. documented that doxorubicin-loaded PEVs (doxo-PEVs) were taken by HL60, K562 cells (leukemia cell lines), and blast cells, in whole blood harvested from patients with newly diagnosed leukemia. Doxo-PEVs were uptaken by cells via P-selectin ligands and integrins. Moreover, doxo-PEVs transfer into leukemia cells was higher, compared to free doxorubicin, which could be used to increase the effectiveness of the therapy and minimize the side effects of drugs [56]. Gasperi et al. showed that PEVs with miR-126 and with miR-223 increased sensitivity of BT549 cells to the cisplatin chemotherapy [62].

PEVs drug-loaded could be a natural vectors-targeted medications. Engineering them from autologous platelets in large quantity and storing for several days, seems to be a new biocompatible and non-immunogenic new-generation medicine. However, to make PEVs applicable and efficacious in clinical treatments, some of their underlying functions still need to be better researched and understood.

## 7. Summary

PEVs biogenesis depends on different signals that control their formation from PLTs. The role of PEVs in various physiological conditions, like hemostasis, or pathological like inflammation or atherosclerosis was confirmed. This review focused on the PEVs participation in cancerogenesis. A better understanding of the biology of PEVs and the mechanisms that allow them to function as mediators in cell-to-cell communication in cancer growth, could become a contribution to the development of new therapeutic strategies, which could also be applicable in cancer. Moreover, determining the number of PEVs and their cargo becomes a useful diagnostic marker or prognostic factor for the different clinical stages in a variety of neoplasia. Knowledge about the formation of distinct PEVs types dependent on PLTs activators could lead to the development of specific techniques for PEVs-mediated drug delivery to cancer cells, or to TME, to modulate their immune response or angiogenesis.

## Figures and Tables

**Table 1:** Comparison of the PEVs cargo and their function. Biologically active molecules, receptors, enzymes, chemokines were categorized based on their functions of the PEVs, but there are no discrepancies detailed for some molecules.

	Function or Category Name							
	Clotting	Enzymes	Adhesion Molecules	Bioactive Lipids	Programmed Cell Death	Growth Factors	Chemokines /Cytokines	Immune Response
**PEVs** **Cargo (Ref)**	TF [52,53,54]	12-LO [55]	CD41/61 [56,57,58,59] CD31 [49,59]	PS [60]	caspase-3 [58]	TGF β1 [50]	CXCR4-(PF-4) [57,61]	CD 154 [32,62]
	FVa, FVIII [60,63]	heparynase [64]	CD62P [57,59,65,66]	AA [67,68]	CD95 [57]	PDGF bFGF [64]	IL-1β [69]	C5b-9 [70]
	PAR-1 [57]	PDI [71]	fibrinogen, vWF, vitronectin [65]	LPA [70]	caspase-9 [72]	VEGF [64]	CCL5, CCL23 [50,73]	CD55, CD59 [52]
	TFPI [74]	NADPH oxidase [75]	CD42a, CD42b [49,59]	TXA2 [76]			CX3CR1 [73]	Factor H [52]

**Table 2:** The role of PEVs in cell-to-cell communication. PEVs secreted from activated PLTs transfer to target cells and their cargo promotes phenotypic changes and novel functions in donor cells.

Target Cell	PEVs Derived Factors/Molecules	Functional Changes (References)
A549, CRL 2066, CRL 2062, HTB 183, HTB 177 lung CCL; LCC * CCL	CD41, CD61 CD184	(+) adhesion to fibrinogen and HUVECs [83] (+) metastatic potential [83] (+) mRNA expression of angiogenic factors (MMP-9, VEGF, IL-8) [83] (+) proliferation and chemoinvasion [83]
HUVECs	miRNA Let-7a miRNA-223	(−) synthesis THBS-1 anti-angiogenic molecule [85] (+) apoptosis by IGF-1 [4,87]
MC-38 colon CCL, LCC * CCL	miRNA-24	(+) apoptosis [84]
BT549 breast CCL	miRNA-123 miRNA-233	(−) migration [62] (−) cell cycle [62]
SKOV3 ovarian CCL	miRNA-939	(+) invasion via TPM3 [94] (+) progression [94]
MDM-MB-231 breast CCL		(+) invasion [96] (+) migration [94]
SGC7901 gastric CCL	miRNA-223	(+) proliferation and invasion [4,105] (−) apoptosis [4,105]
PBMCs from patients with ALL	CD95 Caspase-3	(+) apoptosis [100]
Cl-1 prostate CCL	MMP-2 miRNA?	(+) migration [101]
macrophages	PS, gpIIb/IIIa miR-126-3p	polarization into macrophage M2 [97] (−)CCL4, CSF1, TNF [88]
NK cells	miR-183	(−) cytolysis [102]

Abbreviations: CCL-cancer cell line; * murine cell line; (+) increase; (−) decrease.

## Abbreviations

ABs	apoptotic bodies
ALL	acute lymphoblastic leukemia
CD	cluster of differentiation
CCL	cancer cell line
CLEC-2	C-type lectin domain family 1-member B
ECTSMs	ectosomes
ESCRT	endosomal sorting complex required for transport
ESE	early sorting endosomes
EVs	extracellular vesicles
EXSMs	exosomes
HAECs	human aortic endothelial cells
HUVECs	human umbilical vein endothelial cells
ILVs	intraluminal vesicles
LA	lactadherin
LAMP-1	lysosome-associated glycoprotein-1
LPS	lipopolysaccharide
LXA4	lipoxin A4
MAPK	mitogen-activated protein kinase
Mk-EVs	EVs derived from megakaryocytes
MMP	metalloproteinase
MVBs	multivesicular bodies
MPs	microparticles
NSCLC	non-small cell lung cancer
OSCC	oral squamous cell carcinoma
PBMCs	peripheral blood mononuclear cells
PdEXSMs	platelet derived exosomes
PEVs	platelets extracellular vesicles
PLTs	platelets
PMPs	platelets microparticles
PUFAs	polyunsaturated fatty acids
RT	room temperature
TEVs	tumor derived extracellular vesicles
TF	tissue factor
TGF-β1	transforming growth factor beta 1
THBS-1	thrombospondin-1
TLR-4	toll-like receptor 4
TME	tumor microenvironment
TPM3	tropomyosin 3
VEGF	vascular endothelial growth factor
vWf	von Willebrand factor
